# Allele burden expansion in *JAK2* ^V617F^ and *CALR*‐mutated myeloproliferative neoplasms predicts disease progression and is associated with anagrelide treatment

**DOI:** 10.1002/hem3.70460

**Published:** 2026-08-31

**Authors:** Zi Yun Ng, Laurane Cottin, Marine Demoy, Damien Luque Paz, Corentin Orvain, Wayne‐Corentin Lambert, Jean‐Christophe Ianotto, Eric Lippert, David M. Ross, Diana Iarossi, Alesia Khan, Daniel Lock, Catherine Truong, Kamel Laribi, Yun Fan Yang, Laura Li Gagnon, Valerie Ugo, Claire N. Harrison, Jennifer O'Sullivan

**Affiliations:** ^1^ Department of Haematology Guy's and St Thomas' NHS Foundation Trust London United Kingdom; ^2^ Department of Haematology Royal Perth Hospital Perth Western Australia Australia; ^3^ Department of Haematology Rockingham General Hospital Perth Western Australia Australia; ^4^ Univ Angers, Nantes Université, CHU Angers Inserm, CNRS, CRCI2NA Angers France; ^5^ Service d'Hématologie biologique Hôpital de la Cavale Blanche, CHU de Brest Brest France; ^6^ Service d'Hématologie et d'Hémostase clinique Institut de Cancérologie et Hématologie, CHU de Brest Brest France; ^7^ Department of Haematology Royal Adelaide Hospital and Flinders Medical Centre Adelaide South Australia Australia; ^8^ Haematological Malignancy Diagnostic Service Leeds Teaching Hospital NHS Trust Leeds United Kingdom; ^9^ Hématologie Clinique, CH Le Mans Le Mans France; ^10^ Department of Haematology, West China Hospital Sichuan University Chengdu Sichuan China; ^11^ Department of Haematology CHU de Québec‐Université Laval Quebec Canada; ^12^ Medical Research Council (MRC) Weatherall Institute of Molecular Medicine University of Oxford Oxford United Kingdom

Recent prospective studies demonstrated that reductions in *JAK2* ^V617F^ variant allele frequency (VAF) correlate with clinical benefit in essential thrombocythemia (ET) and polycythemia vera (PV).^1^, ^2^ However, the impact of *CALR* VAF dynamics with therapy on clinical outcomes remains relatively unknown. Pegylated interferon‐alpha (IFN) has been shown to variably reduce *CALR* VAF, but changes with treatments such as hydroxycarbamide (HC), anagrelide (ANA), and ruxolitinib (RUX), a JAK inhibitor, are less well described.^3^ Newer mutation‐targeted therapies are demonstrating early significant reductions in VAFs, which may serve as a surrogate marker of disease modification.^4^

We therefore sought to evaluate dynamics of driver mutation VAF in myeloproliferative neoplasm (MPN) patients treated with cytoreductive therapies and correlate these with clinical outcomes. Sequential cases were collated from eight international centers including the prospective MAJIC‐ET study.^5^ Although MAJIC‐ET has been reported, inclusion enabled greater power for time‐dependent outcomes. A comparator cohort of *JAK2* ^V617F^‐mutated patients was included; the MAJIC‐PV cohort was excluded due to substantially higher baseline VAF precluding meaningful comparison. *CALR* VAF was quantified using next‐generation sequencing (NGS), fragment analysis, or droplet digital polymerase chain reaction (ddPCR). *JAK2* ^V617F^ VAF was quantified using quantitative PCR or NGS. Serial VAF measurements within each patient were performed using the same analytical platform at the same center throughout follow‐up. Molecular response (MR), comprising complete (CMR) and partial (PMR) response as defined per IWG‐MRT/ELN 2013 criteria, plus an exploratory PMR20 category, is detailed in Supporting Information S2: Supplementary Methods.^6^, ^7^, ^8^

Overall, 347 patients with *CALR* (*n* = 190) and *JAK2* ^V617F^ (*n* = 157) mutations with VAF measurements at two timepoints at least 6 months apart were included (Table 1). ET was the most prevalent diagnosis (*n* = 235, 67.7%), followed by primary and secondary myelofibrosis (MF; *n* = 73, 21.1%), prefibrotic myelofibrosis (*n* = 17, 4.9%), PV (*n* = 15, 4.3%), MPN‐unclassified (*n* = 6, 1.7%), and blast‐phase MPN (*n* = 1, 0.3%). Treatment was defined as the predominant cytoreductive agent active between the first and second VAF measurements (Supporting Information S2: Supplementary Methods). Treatments included IFN (*n* = 132, 38.0%), RUX (*n* = 70, 20.2%), ANA (*n* = 29, 8.4%), HC (*n* = 88, 25.4%), and other therapies (*n* = 28, 8.0%) (Table 1). Median VAF measurement interval was 54 months (range 6–246), median follow‐up 123 months (range 8–469), and median treatment duration was 55 months (range 4–289).

**Table 1:** *JAK2* ^*V617F*^ */CALR*‐mutated patient baseline clinical and mutation characteristics.

	*JAK2* ^V617F^‐mutated, *n* = 157 *n* (%)	*CALR*‐mutated, *n* = 190 *n* (%)	P*‐*value
Median age at diagnosis in years (range)	56 (18–90)	55 (9–91)	0.59
Male gender	73 (46.5)	106 (55.8)	0.09
MPN diagnosis			<0.0001
• PV	15 (9.6)	N/A	
• ET	120 (76.4)	115 (60.5)	
• Prefibrotic MF	3 (1.9)	14 (7.4)	
• Primary and secondary MF	17 (10.8)	56 (29.5)	
• MPN‐NOS	2 (1.3)	4 (2.1)	
• Blast‐phase MPN	–	1 (0.5)	
Treatment			0.15
• Interferon‐α^a^	67 (42.7)	65 (34.2)	
• Ruxolitinib	33 (21.0)	37 (19.5)	
• Hydroxycarbamide	37 (23.6)	51 (26.8)	
• Anagrelide	13 (8.3)	16 (8.4)	
• Others	7 (4.4)	21 (11.1)	
Duration of treatment in months, median (range)	72.6 (5–284)	60.0 (4–289)	0.01
Prior treatment lines			0.58
• 0	60 (38.2)	69 (36.3)	
• 1	53 (33.8)	58 (30.5)	
• ≥2	44 (28.0)	63 (33.2)	
Prior anagrelide	30 (19.1)	30 (16.5)	0.53
*CALR* type	N/A		N/A
• Type 1/like		112 (59.0)	
• Type 2/like		60 (31.6)	
• Atypical		18 (9.4)	
Baseline VAF in %, median (range)	20.0 (0.2–95.0)	37.8 (7–65)	0.0001
NGS performed	108 (68.8)	157 (82.6)	
Additional mutation	44 (40.7)	76 (48.4)	
• *TET2*	18 (11.5)	26 (13.7)	
• *ASXL1*	10 (9.2)	17 (4.4)	
• *DNMT3A*	10 (9.2)	7 (4.4)	
• *EZH2*	3 (2.8)	4 (2.5)	
• *IDH1/IDH2*	1 (0.9)	2 (1.3)	
HMR mutation	15 (9.6)	20 (10.5)	0.76
Thrombosis	40 (25.5)	29 (15.6)	0.02
Hemorrhage	13 (8.3)	20 (11.0)	0.40
Disease progression	35 (22.3)	28 (14.7)	0.07
• MF	25 (71.4)	19 (67.9)	
• AP/BP‐MPN	3 (8.6)	9 (32.1)	
• MDS/CMML	4 (11.4)	0 (0.0)	
• ET to PV	3 (8.6)	N/A	
Death	17 (10.8)	28 (14.7)	0.28

Abbreviations: AP/BP‐MPN, accelerated phase/blast phase myeloproliferative neoplasm; ET, essential thrombocythemia; HMR, high molecular risk mutations (*ASXL1*, *SRSF2*, *EZH2*, *IDH1*, *IDH2*, and *U2AF1*); MDS/CMML, myelodysplastic syndrome/chronic myelomonocytic leukemia; MF, myelofibrosis; MPN‐NOS, myeloproliferative neoplasm‐not otherwise specified; N/A, non‐applicable; NGS, next‐generation sequencing; PV, polycythemia vera; VAF, variant allele frequency.

^a^ Predominantly pegylated‐IFN‐α2a, three patients received ropeg‐IFN‐α2b.

Median baseline VAF was 20% (0.2–95.0) for *JAK2* ^V617F^ and 37.8% (7–65.0) for *CALR*. Baseline VAF ≥ 50% was more common in *JAK2* ^V617F^‐mutated patients (*n* = 26/157, 16.6%) than *CALR*‐mutated patients (*n* = 12/190, 6.3%), P = 0.004. Median change in VAF (ΔVAF) across the cohort was +0.4% (range −65.95% to +64%) (Figure 1A).

**Figure 1:**
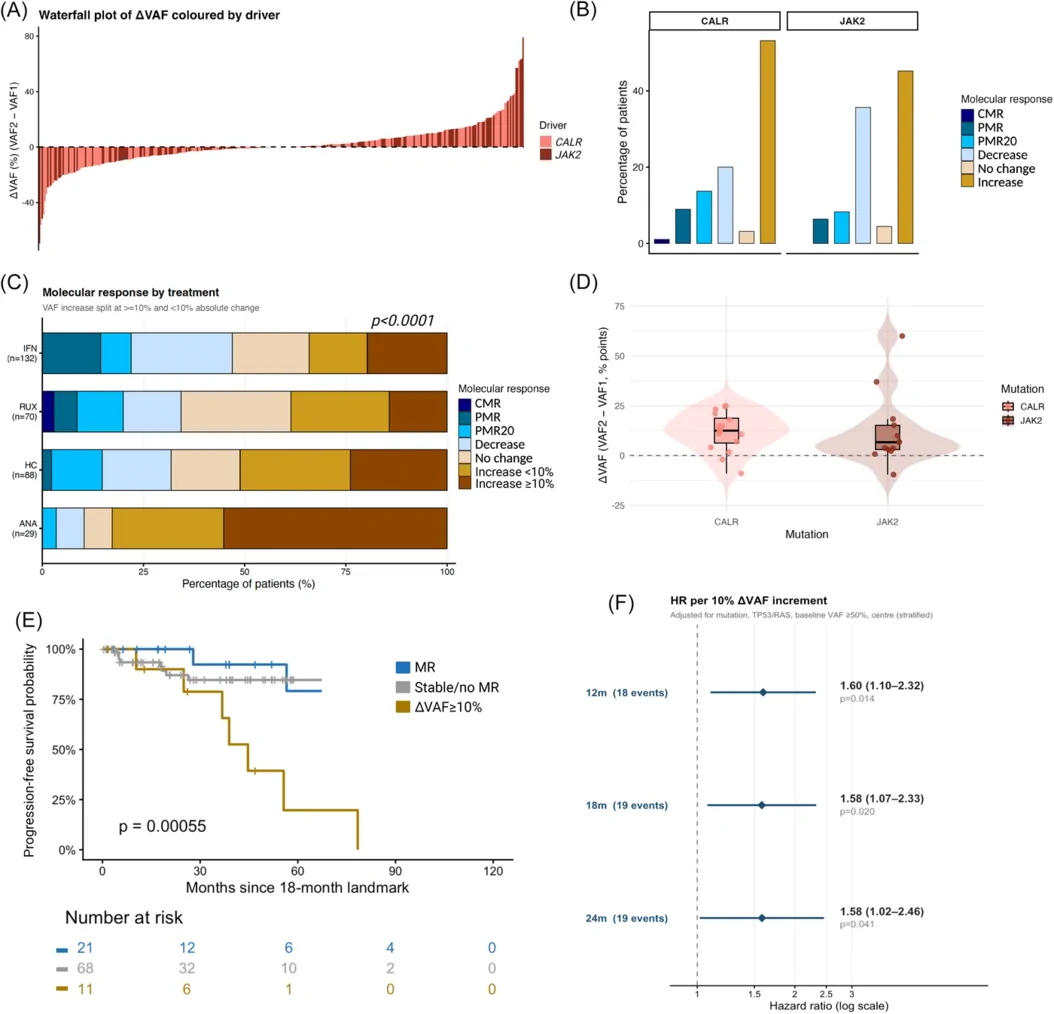
**(A) Waterfall plot showing percentage change in driver mutation variant allele frequency (ΔVAF; VAF2 − VAF1) between the first and second molecular assessments for individual patients, ordered from greatest decrease to greatest increase.** Bars are colored by driver mutation (*CALR* vs. *JAK2* ^*V617F*^). **(B)** Barchart showing distribution of molecular response (MR) categories by driver mutation. **(C)** Stacked barchart showing distribution of MR categories by treatment type. VAF increase colored according to an increase of <10% or ≥10%. P‐value from chi‐squared test across treatment groups. Interferon (IFN) achieved the highest rate of deep MR (complete molecular response [CMR] + partial molecular response [PMR] 14.5%), followed by ruxolitinib (RUX) (8.6%), hydroxycarbamide (HC) (2.3%), and anagrelide (ANA) (0%). **(D)** Violin and boxplot showing absolute change in VAF (ΔVAF) between first and second measurements by driver mutation type (*CALR* vs. *JAK2* ^*V617F*^) in anagrelide‐treated patients. **(E)** Kaplan–Meier curve from 18‐month landmark analysis showing progression‐free survival (PFS) stratified by MR, stable/no MR or allele burden expansion (ΔVAF ≥ 10%). Numbers at risk shown below. **(F)** Forest plot showing hazard ratio per 10% absolute ΔVAF increase from multivariable Cox model at 12‐, 18‐, and 24‐month landmarks, adjusted for driver mutation type, *TP53/RAS* mutations, baseline VAF ≥ 50% and stratified by center. HR, hazard ratio; PMR20, 20%–49% reduction in allele burden.

Non‐MPN driver mutations (NDMs) were present in 33.7% (*n* = 117/347), most commonly *TET2* (*n* = 44, 12.7%) and *ASXL1* (*n* = 27, 7.7%) (Table 1). High molecular risk (HMR) (defined by MIPSS70+),^9^ *RAS* pathway, and *TP53* mutations were identified in 10.1% (*n* = 35), 2.0% (*n* = 7), and 2.8% (*n* = 10) of patients, respectively. NDM data were available at a single timepoint in most patients, precluding longitudinal analysis of clonal evolution. MR comprised CMR in 2 patients (0.6%), PMR in 27 patients (7.8%), and PMR20 in 39 patients (11.2%). Non‐MR decreases, no change (0%–1%), and increases in VAF occurred in 27.1% (*n* = 94), 3.7% (*n* = 13), and 49.5.0% (*n* = 172) of patients, respectively. MRs were similar between *CALR*‐ and *JAK2* ^V617F^‐mutated patients (23.7% vs. 14.6%), though *JAK2* ^V617F^‐mutated patients more often showed VAF decrease without meeting MR criteria (35.7% vs. 20.0%; P = 0.016, Figure 1B). Both CMRs occurred in *CALR*‐mutated ET patients treated with RUX within MAJIC‐ET (*n* = 41 RUX‐treated); RUX was used in only three ET patients at other centers and was predominantly used in MF patients. MR was associated with achieving complete hematological response (68.7% vs. 54.8% in non‐MR patients, P = 0.02) but not with age at diagnosis, MPN subtype, disease duration, or number of prior treatment lines in this dataset.

VAF increase (defined as an absolute increase of ≥10%) occurred in 55.2% of ANA‐treated patients compared with 23.9%, 19.7%, and 14.3% of HC‐, IFN‐, and RUX‐treated patients, respectively (P < 0.0001, Figure 1C). ANA was independently associated with ΔVAF ≥ 10% after adjustment for time between VAF sampling and treatment duration (odds ratio [OR] 5.48, 95% confidence interval [CI] 2.4–12.8; P < 0.001). This association between ANA and ΔVAF ≥ 10% was independent of driver mutation type (P = 0.08, Figure 1D) and was retained when the analysis was restricted to ET patients (P = 0.01, Supporting Information S1: Figure 1A).

We next evaluated whether MR and VAF expansion were associated with clinical outcomes. Disease progression was defined by ELN/IWG‐MRT 2013 criteria.^7^ Overall, 18.2% (*n* = 63) experienced a progression event (Table 1). Overall survival (OS) and progression‐free survival (PFS) (Supporting Information S2: Supplementary Methods) did not differ significantly by MPN subtype in this cohort (Supporting Information S1: Figure 1B,C), although a trend towards inferior OS in MF compared to ET was observed (hazard ratio [HR] 1.85, 95% CI 0.97–3.54, P = 0.063). The attenuation of this expected difference likely reflects selection bias, as MF patients were required to be alive and on cytoreductive therapy with serial VAF monitoring; median OS was not reached in the MF subgroup at a median follow‐up of 123 months (10‐year OS 79.8%), which exceeds typical MF cohorts. Kaplan–Meier estimates demonstrated cumulative progression rates of 13.9% (95% CI 9.8%–17.9%) at 5 years and 31.4% (95% CI 24.0%–38.2%) at 10 years, comparable to published MPN cohorts.

Landmark analyses at 12, 18, and 24 months from the second VAF assessment ensured the VAF change category was assigned prior to outcome, avoiding immortal time bias. Only event‐free patients at each landmark time were included. At the 18‐month landmark (*n* = 100; 19 events), patients with ΔVAF ≥ 10% had subsequent inferior PFS compared with those achieving MR or stable VAF (log‐rank P = 0.000 55, Figure 1E). We next analyzed ΔVAF as a continuous variable in a multivariable Cox model, which demonstrated that each 10% absolute increase in VAF was independently associated with inferior PFS (HR 1.58, 95% CI 1.07–2.33; P = 0.020), with consistent results across the 12‐month (HR 1.60, 95% CI 1.10–2.32; P = 0.014) and 24‐month (HR 1.58, 95% CI 1.02–2.46; P = 0.041) landmarks (Figure 1F). Supporting Information S1: Figure 1D illustrates the graded increase in progression–probability relationship with rising VAF. The model was adjusted for driver mutation type, baseline VAF ≥ 50%^10^, ^11^ (due to its established association with myelofibrotic progression) and *TP53* and *RAS* mutations (combined variable due to low individual event counts and their shared adverse prognostic impact). Other HMR mutations were not associated with progression on univariable analysis and were excluded to avoid overfitting. Center was included as a stratifying variable to account for inter‐center differences in patient selection, treatment practice, and sequencing platform (see Supporting Information S2: Supplementary Methods). Results were consistent in a sensitivity analysis further consolidating the smallest center stratum (HR 1.60, 95% CI 1.08–2.36, P = 0.019 vs. HR 1.58, 95% CI 1.07–2.33, P = 0.02 in the primary model). Findings were consistent across all strata, arguing against center‐specific or sequencing platform‐driven effect.

Importantly, this association was preserved when the analysis was restricted to patients with ET, indicating that VAF expansion confers adverse prognostic significance even in more indolent MPN (Supporting Information S1: Figure 1E,F). The association between ΔVAF ≥ 10% and inferior PFS was not influenced by measurement interval. We evaluated this interval to assess whether VAF testing may have been prompted by clinical suspicion of progression. The second VAF assessment preceded progression by a median of 44.9 months (interquartile range [IQR] 37.5–56.2) in the ΔVAF ≥ 10% group and 29 months (IQR 11.3–42.6) in the ΔVAF < 10% group. Of the 19 progression events at the 18‐month landmark, 5 (26%) occurred within 2 years of the second VAF measurement and 14 (74%) beyond 2 years, further supporting that VAF expansion preceded rather than coincided with clinical disease evolution. No associations were observed in this dataset between thrombotic or hemorrhagic events and VAF dynamics or additional mutations.

In this large multi‐center analysis, MRs were observed across all cytoreductive therapies, most commonly with IFN and RUX, consistent with prior data. We provide novel evidence that driver VAF expansion is independently associated with poorer PFS, even after adjustment for baseline clone size and high‐risk mutations such as *TP53/RAS*. This is in line with previous studies in PV where every 1% increase in VAF translated to a 4.2% increase in adverse events.^1^ Whether VAF expansion is causal or a biomarker of broader disease evolution cannot be established from observational data alone.

Furthermore, we observe for the first time that ANA treatment was associated with a VAF expansion regardless of driver mutation type. This finding aligns with prior data linking ANA treatment to fibrotic transformation, as shown in PT1,^12^ EXELS,^13^ and Mayo Clinic cohorts.^14^ VAF expansion may represent a molecular correlate of the recognized fibrotic risk associated with ANA.

Limitations of our study include non‐uniform VAF measurement intervals and the use of different assay platforms across centers, although this is reflective of current real‐world practice. Nonetheless, all serial measurements per patient were performed on the same platform ensuring changes observed reflect biological variation rather than inter‐assay technical variation. Second, while the association between ANA exposure and VAF expansion was robust, event numbers were insufficient to directly link this molecular effect to fibrotic transformation or survival outcomes in ANA‐treated patients. Third, the absence of an untreated comparator cohort is acknowledged; however, serial VAF monitoring is not performed routinely in the absence of cytoreductive therapy in clinical practice, precluding meaningful comparison with an untreated group in a real‐world cohort.

In summary, our data provide compelling evidence that allele burden expansions (ΔVAF ≥ 10%) represent a meaningful biomarker of disease evolution in clinical practice and may inform treatment decisions. Whilst the benefit of VAF reduction still requires definitive validation in prospective studies, especially in patients receiving novel CALR‐targeted or other mutation‐directed therapies, failure to achieve VAF reduction or subsequent VAF expansion may identify patients who warrant closer monitoring or consideration of alternative therapeutic strategies. Furthermore, the observed association between ANA and VAF expansion supports its positioning below alternative cytoreductive therapies with superior molecular modification potential, and serial VAF monitoring may serve as an early indicator of suboptimal disease control in this setting.

## AUTHOR CONTRIBUTIONS

**Zi Yun Ng**: Conceptualization; writing—original draft; writing—review and editing; formal analysis; project administration; data curation; methodology; software. **Laurane Cottin**: Data curation; writing—review and editing. **Marine Demoy**: Data curation; writing—review and editing. **Damien Luque Paz**: Data curation; writing—review and editing. **Corentin Orvain**: Data curation; writing—review and editing. **Wayne‐Corentin Lambert**: Data curation; writing—review and editing. **Jean‐Christophe Ianotto**: Writing—review and editing; data curation. **Eric Lippert**: Data curation; writing—review and editing. **David M. Ross**: Writing—review and editing; data curation. **Diana Iarossi**: Data curation. **Alesia Khan**: Writing—review and editing; data curation. **Daniel Lock**: Data curation. **Catherine Truong**: Data curation; writing—review and editing. **Kamel Laribi**: Writing—review and editing; data curation. **Yun Fan Yang**: Writing—review and editing; data curation. **Laura Li Gagnon**: Writing—review and editing; data curation. **Valerie Ugo**: Writing—review and editing; data curation. **Claire N. Harrison**: Supervision; writing—review and editing; conceptualization. **Jennifer O'Sullivan**: Supervision; writing—review and editing; writing—original draft; software; formal analysis; data curation; conceptualization; methodology; visualization.

## CONFLICT OF INTEREST STATEMENT

Z.Y.N. has received honoraria from GSK. D.M.R. has received research funding from Novartis and served as a speaker or on advisory panels for Novartis, GSK, Takeda, Merck, Menarini Stemline, Prelude, and Jubilant. C.N.H. has received research funding from Novartis, GSK, and Constellation; she has served as a speaker and on advisory panels for Novartis, AOP, GSK, BMS, Menarini Stemline, Karyopharm, JNJ, Kartos, and Incyte. J.O. has received honoraria from Novartis, Karyopharm, and Menarini Stemline and has served as a speaker for Novartis.

C.N.H. is an Editor of *HemaSphere*.

## ETHICS STATEMENT

This research was conducted in accordance with the Guy's Cancer Cohort Ethics (23/NW/0105).

## FUNDING

FIMBANK was funded by the French “Institut National du Cancer” (INCa BCB 2013 & 2022).

## Supporting information


**Supplementary Figure.** (A) Stacked barchart showing distribution of molecular response categories by treatment type in essential thrombocythemia (ET) patients only. Variant allele frequency (VAF) increase is split at the ≥10% absolute threshold. P‐value from chi‐squared test across treatment groups. (B) Kaplan–Meier (KM) plot showing overall survival by myeloproliferative neoplasm (MPN) subtype, ET, myelofibrosis (MF), prefibrotic myelofibrosis (pPMF), and polycythemia vera (PV). (C) Progression‐free survival (PFS) by MPN subtype, defined as time to transformation to MF, accelerated‐ or blast‐phase disease, myelodysplastic syndrome/chronic myelomonocytic leukemia, or death. (D) Logistic regression curve showing the continuous relationship between absolute ΔVAF and probability of disease progression at the 18‐month landmark (*n* = 100, 19 events). Individual patients are shown as jittered points (0 = no progression, 1 = progression). The dotted vertical line indicates the absolute increase of VAF ≥ 10% (ΔVAF ≥ 10%) threshold. Despite only *n* = 11 patients with ΔVAF ≥ 10%, 7/11 (63.6%) progressed compared with 12/89 (13.5%) in the ΔVAF < 10% group. (E) Kaplan–Meier curve for progression‐free survival from the 18‐month landmark in ET patients only, stratified by molecular response (MR), stable/no MR, or allele burden expansion (ΔVAF ≥ 10%). Log‐rank P = 0.0037. Numbers at risk shown below. (F) Forest plot showing hazard ratio per 10% absolute ΔVAF increment from multivariable Cox regression at 12‐, 18‐, and 24‐month landmarks in ET patients only, adjusted for driver mutation type, *TP53/RAS* mutations and baseline VAF ≥ 50%, stratified by center. ΔVAF, absolute change in variant allele frequency; ANA, anagrelide; CI, confidence interval; CMR, complete molecular response; HC, hydroxycarbamide; HR, hazard ratio; IFN, pegylated interferon‐alpha; MPN, myeloproliferative neoplasm; PMR, partial molecular response; PMR20, 20%–49% reduction in allele burden; RUX, ruxolitinib.

Supporting File 1.

## Data Availability

The data that support the findings of this study are available on request from the corresponding author. The data are not publicly available due to privacy or ethical restrictions.
