# Supplementary material for: Allele burden expansion in JAK2 V617F and CALR‐mutated myeloproliferative neoplasms predicts disease progression and is associated with anagrelide treatment
Source: Hemasphere. 2026 Aug 31;10(9):e70460. doi: 10.1002/hem3.70460 (PMC13527725; doi:10.1002/hem3.70460)
Supplement: Supplementary file 1 [file HEM3-10-e70460-s001.pdf]

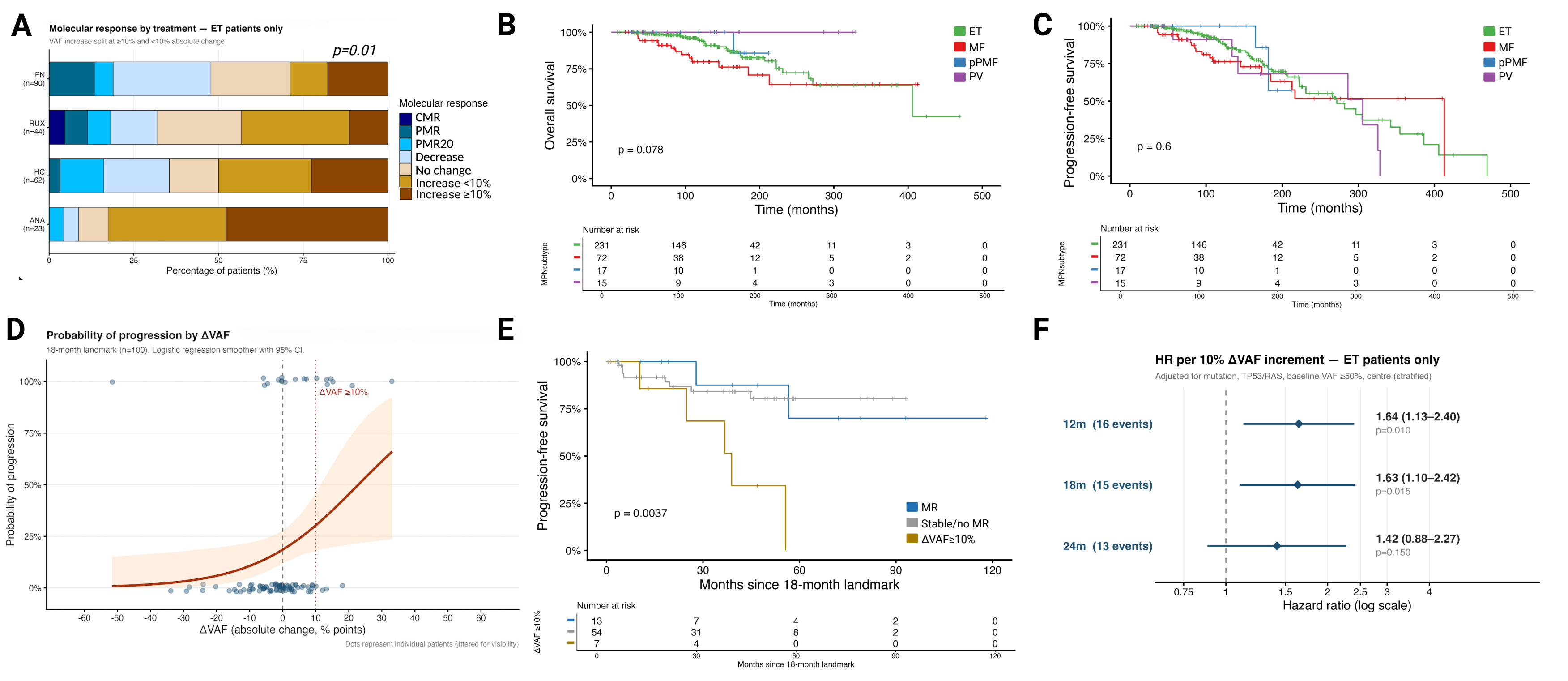
A
Molecular response by treatment — ET patients only
VAF increase split at ≥10% and <10% absolute change
p=0.01
IFN (n=90)
RUX (n=44)
HC (n=62)
ANA (n=23)
Molecular response
CMR
PMR
PMR20
Decrease
No change
Increase <10%
Increase ≥10%
0 25 50 75 100
Percentage of patients (%)
B
Overall survival
100% 75% 50% 25% 0%
p = 0.078
ET
MF
pPMF
PV
0 100 200 300 400 500
Time (months)
Number at risk
MPNsubtype
231 146 42 11 3 0
72 38 12 5 2 0
17 10 1 0 0 0
15 9 4 3 0 0
Time (months)
C
Progression-free survival
100% 75% 50% 25% 0%
p = 0.6
ET
MF
pPMF
PV
0 100 200 300 400 500
Time (months)
Number at risk
MPNsubtype
231 146 42 11 3 0
72 38 12 5 2 0
17 10 1 0 0 0
15 9 4 3 0 0
Time (months)
D
Probability of progression by ΔVAF
18-month landmark (n=100). Logistic regression smoother with 95% CI.
Probability of progression
100% 75% 50% 25% 0%
ΔVAF ≥10%
-60 -50 -40 -30 -20 -10 0 10 20 30 40 50 60
ΔVAF (absolute change, % points)
Dots represent individual patients (jittered for visibility)
E
Progression-free survival
100% 75% 50% 25% 0%
p = 0.0037
MR
Stable/no MR
ΔVAF≥10%
0 30 60 90 120
Months since 18-month landmark
Number at risk
ΔVAF ≥10%
13 7 4 2 0
54 31 8 2 0
7 4 0 0 0
Months since 18-month landmark
F
HR per 10% ΔVAF increment — ET patients only
Adjusted for mutation, TP53/RAS, baseline VAF ≥50%, centre (stratified)
12m (16 events) 1.64 (1.13–2.40) p=0.010
18m (15 events) 1.63 (1.10–2.42) p=0.015
24m (13 events) 1.42 (0.88–2.27) p=0.150
0.75 1 1.5 2 2.5 3 4
Hazard ratio (log scale)
