# Supplementary material for: Allele burden expansion in JAK2 V617F and CALR‐mutated myeloproliferative neoplasms predicts disease progression and is associated with anagrelide treatment
Source: Hemasphere. 2026 Aug 31;10(9):e70460. doi: 10.1002/hem3.70460 (PMC13527725; doi:10.1002/hem3.70460)
Supplement: Supplementary file 2 [file HEM3-10-e70460-s002.docx]

**Supplementary Methods**

**Treatment assignment validation**

To validate the approach taken to define the predominant cytoreductive agent, we confirmed that 91.3% of patients remained on their predominant treatment at the time of the second VAF measurement, and 87.3% and 76.3% had been on this treatment for at least 6 and 12 months respectively prior to second VAF, indicating that observed VAF changes reflect sustained exposure to the assigned agent. Median duration on the predominant treatment prior to second VAF was 49 months for IFN, 54.5 months for HC, 39 months for ANA and 17.5 months for RUX. Where patients received more than one treatment during the VAF interval, the treatment accounting for the greatest proportion of that period was assigned; the proportion of the VAF interval covered by the predominant treatment had a median of 86.9%, with 78% of patients having ≥75% of their VAF interval covered by a single agent.

**Molecular response definitions**

Molecular response (MR) was defined as per IWG-MRT/ELN 2013 criteria.^1,2^ Complete molecular response (CMR) was defined as eradication of a preexisting abnormality, and partial molecular response (PMR) as a ≥50% relative decrease in VAF in patients with at least 20% VAF at baseline. An exploratory endpoint, "PMR20", defined as a 20–49% relative decrease in VAF, was included as it has been shown to correlate with clinical/histological response and overall survival in clinical trials.^3^

**Statistical analysis**

Clinical characteristics were compared between molecular and treatment groups using Pearson’s chi-squared test for categorical variables and Wilcoxon rank-sum test for continuous variables, as appropriate based on data distribution.

Median follow-up time was estimated using the reverse Kaplan–Meier method, with censoring and event indicators reversed. Progression-free survival (PFS) was defined as the time from study entry to transformation to myelofibrosis, accelerated- or blast-phase disease, development of myelodysplastic syndrome or chronic myelomonocytic leukemia or death from any cause. Overall survival was defined as time from study entry to death from any cause.

Survival outcomes were estimated using the Kaplan–Meier method and compared using the log-rank test. To mitigate immortal time bias in analyses evaluating the prognostic impact of molecular response and VAF change, landmark analyses were performed at 12, 18, and 24 months following the second VAF assessment. Patients were classified at each landmark into molecular response (any ELN-defined molecular response), stable/no molecular response or VAF increase (ΔVAF ≥10%).

Multivariable survival analyses were conducted using Cox proportional hazards regression. Covariates were selected based on univariable significance and biological plausibility, with inclusion limited by event numbers. Mutations were analyzed both as individual genes and as predefined mutation groups, including high molecular risk (HMR) genes (*ASXL1, SRSF2, EZH2, IDH1, IDH2, U2AF1*). Exploratory analyses additionally evaluated inclusion of *SF3B1* and *NFE2* within the HMR group. Only mutations demonstrating a statistically significant association with outcomes on univariable analysis (*p*<0.05) were retained in multivariable models to minimize overfitting. *TP53* and *RAS* pathway mutations were modelled as a combined variable due to low individual prevalence and shared adverse prognostic impact. Data were contributed from eight centers. Given variation in center size, contributing centers were consolidated into five strata for Cox models (Angers n=126, MAJIC n=75, Brest n=57, GSTT n=52, and a combined 'Other' group incorporating centers contributing fewer than 15 patients, n=30). Center was incorporated as a stratifying variable to account for inter-center differences in patient selection, treatment practice and sequencing platform, allowing each stratum its own baseline hazard while assuming a consistent effect of ΔVAF across sites. Proportional hazards assumptions were assessed using Schoenfeld residuals.

All statistical analyses were performed using R statistical software (R Foundation for Statistical Computing), with relevant packages including *survival* and *coxphf*. Figures were generated using *ggplot2*. De-identified individual patient data are available from the corresponding author on reasonable request.

**References:**

1.	Tefferi A, Cervantes F, Mesa R, et al. Revised response criteria for myelofibrosis: International Working Group-Myeloproliferative Neoplasms Research and Treatment (IWG-MRT) and European LeukemiaNet (ELN) consensus report. *Blood*. Aug 22 2013;122(8):1395–8. doi:10.1182/blood-2013-03-488098

2.	Barosi G, Mesa R, Finazzi G, et al. Revised response criteria for polycythemia vera and essential thrombocythemia: an ELN and IWG-MRT consensus project. *Blood*. 2013;121(23):4778–4781. doi:10.1182/blood-2013-01-478891

3.	Mascarenhas J, Komrokji RS, Palandri F, et al. Randomized, Single-Blind, Multicenter Phase II Study of Two Doses of Imetelstat in Relapsed or Refractory Myelofibrosis. *J Clin Oncol*. Sep 10 2021;39(26):2881–2892. doi:10.1200/jco.20.02864
